# Global tracking of access and quality in early childhood care and education

**DOI:** 10.1186/s40723-023-00116-5

**Published:** 2023-05-02

**Authors:** Abbie Raikes, Nirmala Rao, Hirokazu Yoshikawa, Caroline Cohrssen, Jere Behrman, Claudia Cappa, Amanda Devercelli, Florencia Lopez Boo, Dana McCoy, Linda Richter, the UKRI GCRF Harnessing the power of global data to support young children’s learning and development research group

**Affiliations:** 1 College of Public Health, University of Nebraska Medical Center, Omaha, USA; 2 Faculty of Education, The University of Hong Kong, Hong Kong, Hong Kong; 3 Global TIES, New York University, New York, USA; 4 University of New England, Armidale, Australia; 5 University of Pennsylvania, Philadelphia, USA; 6 UNICEF, New York, USA; 7 World Bank, Washington, USA; 8 Inter-American Development Bank, Washington, USA; 9 Harvard University, Cambridge, USA; 10 University of Witswatersand, Johannesburg, South Africa

**Keywords:** Early childhood care and education, Definition, Preprimary education, National-level monitoring and measurement, SDG Target 4.2

## Abstract

Investments in early childhood care and education (ECCE) have contributed to a growing demand for internationally comparable data. Yet data on access to quality ECCE are not routinely collected in many countries, leading to limited information on equitable access to ECCE, quality of provision, and the impact on learning and wellbeing outcomes. This paper outlines the current status of global measurement of access to quality ECCE and identifies issues with definitions, availability, and accuracy of ECCE data across countries and outlines paths forward. We argue that estimates of access to ECCE should be based on children’s participation in quality ECCE across multiple program types, rather than enrollment or attendance alone, given the critical importance of dosage and participation for ensuring positive benefits from ECCE. Governments, international organizations, and researchers all have roles to play in setting standards to define and monitor ECCE, generating workable tools for measuring nationally, and globally investing in national monitoring systems and routine household surveys to obtain accurate estimates of access to quality ECCE.

Providing children with the best possible start to life is a growing priority across nations. Therefore, there is a push for internationally comparable data to track changes and equity in young children’s access to quality early childhood programs within and across countries. Recent reviews of evidence from high income countries (HICs) as well as low- and middle-income countries (LMICs) demonstrate that participation in high-quality early childhood care and education programs can lead to lasting positive impacts on child development (Atteberry et al., 2019; Berlinski, et al., 2009; Heckman et al., 2010; Ludwig & Miller, 2007; Onyango et al., 2021; Rao et al., 2014). Globally, the most recent data from UNICEF and UNESCO pre-COVID pandemic from 196 countries showed that ECCE enrollment for the population of children between age 3 and primary school entry was 54% globally, ranging from 21% in low-income countries to 79% in high-income countries (McCoy et al., 2021). Private provision is common, estimated at 37% across all ECCE, and is especially common for children under age three, estimated at 57% in 2018 (UNESCO, 2021).

Although enrollment in formal preprimary programs has shown steady increases over the last decade, access and enrollment in preprimary education are still marked by notable inequities based on region, family income, and other factors (Global Partnership for Education, 2020). Across all countries, children from higher-income families are significantly more likely to attend ECCE, and children in HICs have much greater access than children in low-income countries (McCoy, 2018). Children from poorer households and those from rural areas were less likely to attend ECCE, across 61 countries with available data between 2012 and 2019 (UNESCO, 2021). Further, the COVID pandemic resulted in widespread closures that represented an average of 52 days of lost instruction at the country level (McCoy et al., 2021). While inequity in access to quality programs is found to various degrees in every country, challenges faced by young children LMICs are generally greater than HICs (Chaudry et al., 2020). In 2010, 43% of children under the age of 5 years in LMICs were estimated to be at risk of suboptimal development with quantifiable economic implications for individual, national, and global losses (Black et al., 2017). Risks to vulnerable young children have been compounded by the impact of the COVID-19 pandemic, especially regarding access to quality early childhood care and education (Lopez Boo et al., 2020; McCoy et al., 2021; Yoshikawa et al., 2020). An estimated 40% of the world’s young children—or 350 million children birth to school entry—lack access to quality childcare, further worsening family poverty by limiting parental employment and increasing parental stress (Devercelli & Beaton-Day, 2020).

This paper focuses on data on access to quality early childhood care and education: having reliable, accurate information is essential for tracking equity in access to quality early childhood programs. Weak data and monitoring systems have been identified as a key barrier in increasing access to quality early childhood programs, in low-resource settings specifically (Shawar & Shiffman, 2017). Acknowledging the range of definitions and acronyms, we use the term “early child development” (ECD) to refer to the maturational processes that take place during the period from conception through the start of formal schooling (Black et al., 2017). The term “early childhood care and education” (ECCE) broadly encompasses the environments that influence children’s holistic developmental processes, including both home environments and out-of-home environments, with attention to children’s social, emotional, physical, and cognitive development (UNESCO, 2016). For the purposes of this paper, we define ECCE programs as organized, non-familial learning, and care programs directly serving children before formal primary school entry, beginning at birth and extending through the start of primary education, and intended to support learning as well to provide care. Reflecting the importance of taking a broad, intersectoral approach to child development that integrates health, nutrition, social protection, and education, early childhood programs often sit within or across multiple governmental ministries, including education, social protection, health, and women’s and children’s affairs.

Although access to quality has been defined as critical in achieving the promise of long-term benefits of ECCE, challenges of definition and measurement remain. These have constrained advances at both program and national policy levels. The goals of this paper are twofold: to describe these definitional and measurement challenges as they result in limited globally comparable data on access to quality ECCE, and thereafter to suggest ways forward.

## Quality in ECCE: an overview

**Fig. 1:**
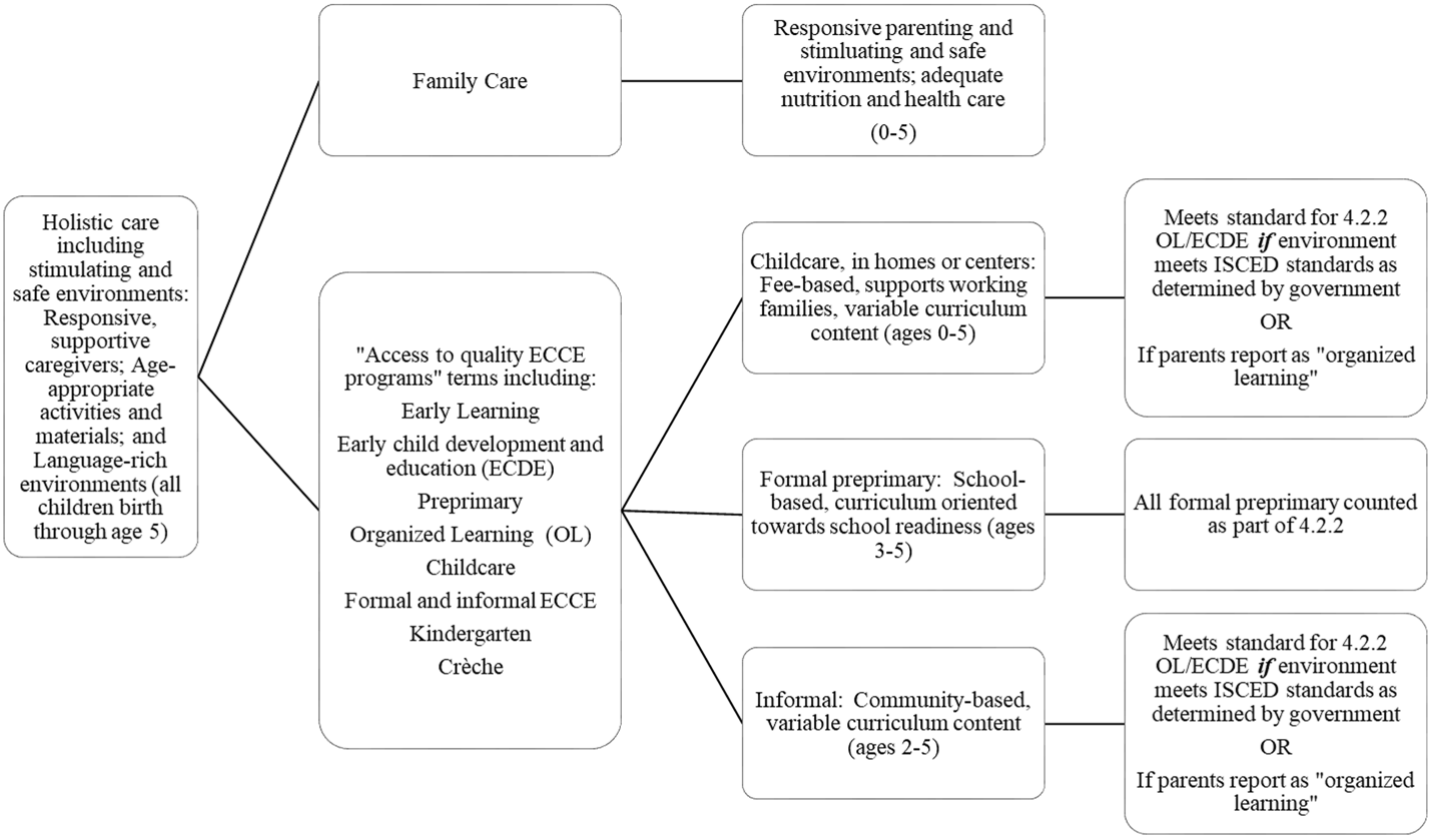
Types of ECCE viewed through the lens of Target 4.2.2: Access to quality ECCE

What counts as a quality ECCE program, among the variety of programs for young children, including fee- and no-fee-based services, informal and formal settings, and community-based settings? Although there have been few systematic, cross-country inventories of early childhood terminology, the ways in which countries describe early childhood programs and systems vary. In some countries, early childhood education (ECE) and early childhood education and care (ECEC) are umbrella terms that include “childcare” (which in some countries is understood to place more emphasis on care than learning) and kindergarten/nursery school (which in some countries is understood to include a more structured focus on learning as well as care) as well as parenting programs delivered through home visiting or group settings. Other countries use early childhood care and development (ECCD) or early childhood development (ECD) to describe services for children ranging in age from three to six years. Preschool Education (PSE) and Pre-Primary Education (PPE) typically refer to more formal early learning services for children ranging in age from three to six years that aim to prepare children for transition to school, though some countries de-emphasize the goal of “school readiness.” A further complexity is the use of the term ECD in some jurisdictions to refer to a *site* of early learning, rather than the *process* of learning and development, during the early years, or to a method of promoting learning and development (e.g., “my child attends ECD”). “Early childhood development” may thus refer to a phase of development, a process of development, and an intervention. Different terms for ECCE programs thus include but are not limited to early childhood education offered at centers or schools or formal preprimaries; community-based preschools and early childhood programs; and in some countries, childcare (also called daycare), crèche, and kindergarten settings (see Fig. 1 for an overview of the range of provision). The focus of this paper is on these various forms of ECCE programs, for succinctness referred to as ECCE from this point forward. This paper is not intended to suggest common terms should be used across all countries and institutions, but rather that it is important to clarify the various terms used and to offer ways forward to better define them in the interest of consistent measurement.

The United Nations Sustainable Development Goals (SDGs) include Target 4.2 which states that by 2030, *all girls and boys have access to quality early child development, care, and preprimary education so that they are ready for primary education*. Measurement plays a key role in attempting to hold countries and global actors accountable for reaching agreed-upon goals for children’s learning (Beeharry, 2021; Raikes et al., 2017). All SGD global indicators were approved by the United Nations Statistical Commission with an accompanying United Nations resolution and are used for the official monitoring of progress toward the SDG targets. UNICEF and the UNESCO Institute for Statistics serve as the custodians for defining measurement and reporting across countries for the indicators under Target 4.2. An additional set of thematic indicators was identified for Target 4.2 by groups of stakeholders convened by UNESCO (Kennedy, 2018; UNESCO, 2017a, 2017b; United Nations Statistical Commission [UNSC], 2021). Target 4.2 is measured by two global and three thematic indicators (see Table 1), most of which are intended to be relevant and comparable across countries, and which can be disaggregated by factors associated with inequity, including but not limited to child sex, place of residence, ethnicity, migration status, disabilities, race/ethnicity, and family income/wealth. A key indicator, percentage of children who are developmentally on track, is measured by UNICEF’s Early Childhood Development Index (ECDI). Other measures of children’s development used in low- and middle-income countries include Save the Children’s IDELA (Pisani et al, 2018), the Measuring Early Learning Quality & Outcomes measures (UNESCO, 2017a, 2017b), the Global Scale for Early Development (World Health Organization [WHO], 2018), and the East Asia–Pacific Child Development Scales (Rao et al., 2019, 2022).

**Table 1:** Global indicators of Target 4.2 on early childhood development

Target and Indicators	
4.2	By 2030, all girls and boys have access to quality early child development, care and preprimary education so that they are ready for primary education
**4.2.1**	**Proportion of children aged 24 and 59 months of age who are developmentally on track in health, learning, and psychosocial wellbeing, by sex**
**4.2.2**	**Participation rate in organized learning (one year before the official primary entry age), by sex**
4.2.3	Percentage of children under 5 years experiencing positive and stimulating home learning environments
4.2.4	Gross early childhood education enrollment ratio in (a) preprimary education and (b) early childhood educational development^a^
4.2.5	Number of years of (a) free and (b) compulsory preprimary education guaranteed in legal frameworks

^a^UNESCO defines the gross enrollment rate (GER) as the “total enrolment in a specific level of education, regardless of age, expressed as a percentage of the eligible official school-age population corresponding to the same level of education in a given school year” (UNESCO, 2009, p. 9). Global indicators that are part of the global monitoring agenda for the Sustainable Development Goals are noted in bold. Thematic indicators, or those that cover a broader range of sectorial priorities but are not part of the official global monitoring agenda, are not noted in bold

Although “quality” is mentioned in the target’s language, no indicators were proposed for measuring the quality of non-home environments for Target 4.2. This is due to the absence of a global definition of quality and the limited data on ECCE quality available from most countries (UNESCO, 2017a, 2017b). There is wide variation across countries in the design, implementation, and monitoring of early childhood programs, and in the definitions of what counts as “quality.” However, to generate internationally comparable data for global indicators, countries must use the same definitions, measures, and implementation protocols when collecting and reporting data. Data on four out of five of the global and thematic indicators of Target 4.2 are collected through two mechanisms: household surveys that ask primary caregivers to respond to questions about their children, and education management information systems that collect data on schools and children. Data for the final indicator, addressing the presence of national frameworks and entitlements to ECCE, are collected through a review of national documents.

The intent of Target 4.2 is to ensure young children’s healthy development, including access to quality formal preprimary education as well as other types of child development programs. The language of Target 4.2 is focused on school readiness, and the target sits within Goal 4 addressing education. Yet a strong body of scientific work emphasizes the importance of holistic approaches to children’s development beginning before birth. A broad interpretation of access to quality child care, development, and educational programs throughout children’s early lives is thus necessary to ensure that the target is implemented in a holistic manner. A further tension emerges between the feasibility of measurement, and the scope and depth required to accurately measure what is important for children’s development. Fully measuring influences on early child development would necessitate large-scale, coordinated measurement, yet limiting measurement too narrowly, based on what is feasible, runs the risk of producing incomplete data on child wellbeing and the risk that the definition of child wellbeing will eventually become dictated by the measure. Finally, as discussed in greater detail below, defining “quality” in ECCE across countries poses unique challenges as well as the potential for unintended, negative consequences. We identify these issues as important influences on determining progress toward Target 4.2.

While Target 4.2 is measured by multiple indicators, in this paper we focus first on Indicator 4.2.2, namely, participation rates in organized learning, defined by “the percentage of children who have access to quality early child development, care and preprimary education in the year before starting primary school,” which includes programs offering a combination of efforts to promote development, education, and care. Thereafter, we turn to issues of defining and monitoring quality (within both the SDG Goal 4 and Target 4.2, but not in the indicators) in tandem with access to ECCE. We outline the present status of global ECCE monitoring, describe issues and challenges that have impeded progress toward reliable country-level ECCE data, and make recommendations for country and global-level actions to improve ECCE data.

## Global monitoring of ECCE: Trends and present efforts

Access to quality ECCE is achieved through a system characterized by governance encompassing private and public settings, finance, and workforce, coordinated both vertically (from the national to local levels) and horizontally (with other sectors, such as nutrition, health, and child protection (Britto et al., 2014). Indeed, what started as a relatively informal sector is increasingly governed by specific national policies and legislation. The adoption of the SDGs in 2015, including Target 4.2, occurred at a time when many countries were expanding investment in ECCE. By 2019, 76 countries had adopted some form of national, multisectoral ECD policy, including ECCE (Vargas-Barón et al., 2022). In recent years, many countries have made notable progress toward expanding access to ECCE, for example, through national provision of at least one year of fee-free preprimary education (Earle et al., 2018). While we are not aware of any comprehensive inventory of country policies on quality in ECCE, a recent survey of 13 countries in sub-Saharan Africa by the Association for the Development of Education in Africa and Together for Early Childhood Evidence (Raikes et al., 2021) indicated that nearly all have ECCE quality standards in place, and about half routinely perform direct observations of both public and private facilities. Early learning and development standards (ELDS) have been developed in many countries (see Ejuu, 2012; Kagan & Kauerz, 2012; Miyahara & Meyers, 2008), providing guidance on the goals of ECCE programs, encompassing a focus on cognitive, social-emotional, language, and physical development. The most comprehensive review dates to 2017 and described ELDS development in 35 low- and middle-income countries (UNICEF, 2017). Workforce systems have been developed to prepare well-trained ECCE workforces: developing and accrediting qualifications, implementing teacher preparation courses, providing in-service teacher professional development, and monitoring service provision to assess and improve the quality of care and education services. These policy advances indicate a movement toward expanded ECCE services and increasingly intentional approaches to early childhood systems.

### Global indicators’ purpose: providing a diagnostic of ECCE system functioning

When taken together, the indicators used to monitor ECCE provide an important window into the overall functioning of ECCE systems, especially as countries focus on scaling up ECCE programs to reach all children. Population-level data, or data that are representative of the population of children living in a given area, are necessary for comparing the relative coverage and impact of various national-level policies and programs intended to improve children’s early care and education. Thus, the Target 4.2 indicators and early childhood systems have reciprocal significance because they track progress on childhood development itself, as well as access to ECCE programs (and for one thematic indicator, quality of stimulation in home environments).

Accurate data on each of these areas can inform policy action for access and quality. Data on child learning and development provide an indication of how ECCE policies are impacting early learning and development. For example, data on access reveal inequities. As noted above, in LMICs, children from low-income families and from minority groups are less likely to access quality ECCE than their more advantaged peers (Lu et al., 2020; McCoy et al., 2018). These data on inequity guide further investment to reduce geographic, population, or other gaps in access to quality ECCE. The presence of legal frameworks can prompt countries with informal ECCE systems to provide consistent policy frameworks that uphold children’s and families’ rights, and to fund them. Together, the set of globally comparable Target 4.2 indicators can provide broad insight into the overall functioning of ECCE. This in turn helps hold key stakeholders accountable for making progress, identifies inequitable access to services, and reveals the impact of ECCE policies and programs across populations.

### Limited and unaligned data on ECCE

Despite expansion of services, data on ECCE are limited, constraining the ability of many countries to build data-driven early childhood policies and programs (Global Partnership for Education, 2019). The data used to report on Indicator 4.2.2 are complex. The SDGs include three data sources for indicators of children’s access to ECCE programs: (1) children’s attendance in organized learning as reported by responses to household surveys of children’s primary caregivers, primarily through UNICEF-supported Multiple Indicator Cluster Survey (MICS) and the USAID-supported Demographic & Health Survey (DHS); (2) governmental administrative data on enrollment in preprimary education, typically school based and potentially not including private ECCE providers who may not be registered in government systems; and (3) access to “early childhood educational development” (a term used by UNESCO and defined in greater detail below). Both 2 and 3 are overseen by the UNESCO Institute for Statistics. The definitions used to generate these sources of data are aligned with the International Standard Classification of Education (ISCED) levels 0.01 and 0.02 which outline standard definitions of access to educational programs in the years before primary school (see Table 2 for specific definitions used by each organization). Ideally, the sources of data that are used to create indicators would be defined in similar ways across surveys and administrative data, for example, children should be enrolled and attend regularly to meet the intent of “access to quality ECCE.” But at present, household surveys and administrative data do not align. This lack of alignment raises many questions. Are children enrolling in ECCE but not attending, in which case, is the intent of Target 4.2 met? Does attendance, as understood by primary caregivers responding to household survey questions, meet standards for quality ECCE? What do we know about children who are five years of age but not yet in formal preprimary education?

**Table 2:** Definitions and methods for data collection on various types of early childhood education

	Ages	How data are collected	Definition
International Standards for Classification of Education (ISCED) 0	Birth to start of primary school	Surveys or national monitoring	0.1: Education designed to support early development in preparation for participation in school and society. Programs designed for children below the age of 3 0.2: Education designed to support early development in preparation for participation in school and society. Programs designed for children from age 3 to the start of primary education
UNESCO^a^ 1) preprimary and 2) early childhood educational development programs		Ministry-reported; dependent on government	Gross early childhood education enrollment ratio in (a) preprimary education and (b) early childhood educational development The educational properties of early childhood educational development are characterized by a learning environment that is visually stimulating and language rich. These programs foster self-expression, with an emphasis on language acquisition and the use of language for meaningful communication. There are opportunities for active play, so that children can exercise their coordination and motor skills under supervision and through interaction with staff. Programs providing only childcare (supervision, nutrition, and health) are not covered by ISCED (see ISCED 2011 Manual, paragraph 105)
UNICEF	Ages 24 months to 59 months	Household surveys	Participation rate in organized learning (one year before the official primary entry age). The participation rate in organized learning (one year before the official primary entry age), by sex as defined as the percentage of children in the given age range who participate in one or more organized learning program, including programs which offer a combination of education and care. Participation in early childhood and in primary education are both included. The age range will vary by country depending on the official age for entry to primary education. Parents are asked to respond to questions on children’s attendance in early childhood education programs

UNESCO relies on ISCED definitions when providing guidance to national ministries on collection and aggregation of early childhood data

Moreover, limited funds for data collection and analyses mean that each definition and each measurement effort must be carefully designed to maximize returns on investments. While both the UNESCO Institute for Statistics and UNICEF provide guidance on reporting for all indicators for Goal 4, countries decide what data to collect and report among the array of hundreds of SDG indicators, leading to uneven data availability across countries and in many cases, impacting on the availability of globally comparable data. As one example, reporting on Target 4.2 is variable across countries (United Nations, 2021) with few, if any indicators reported during the same year by all countries. In 2019, 78 of 246 countries reported to UNESCO on access to ECCE using governmental administrative data counting children’s access to preprimary or early childhood educational programs, and only eight out of 246 countries reported access to ECCE using household survey data (United Nations, 2021). The number of reporting countries had risen to 129 in 2023 (UNESCO, 2023). However, although reporting on Indicator 4.2 (the percentage of children with access to quality ECCE one year before primary schooling) is not comprehensive across countries, it is the most frequently reported indicator for Target 4.2 (Rao et al., 2021). As context, in 2023, only twenty countries out of 246 reported data on Indicator 4.2.1 (the percentage of children developmentally on track), although it is important to note that such data may not change quickly enough to warrant annual collection. Approximately 70 LMICs reported relevant data for Indicator 4.2.1 at some point between 2010 and 2019, whereas 4.2.2 was among the indicators with the most global coverage during the same period (UNESCO, 2019). Accordingly, reporting on “access to quality ECCE” may be one of the more attainable opportunities to gain insights into progress toward Target 4.2 across countries (noting the important limitation of no indicators focused on quality).

The challenges in generating data on ECCE are exacerbated by the many types of programs, modalities, and sectors involved in programs for young children and their families, and the challenges that governments and civil society face in defining and tracking access to, and participation in, various forms of ECCE. There is wide variation in how early childhood programs are counted and monitored at country level, with differences by child age, who provides the services, and the intended goal of the service (OECD, 2015), as well as varying definitions of quality and of what comprises access to quality ECCE. Measuring access to ECCE may be much more challenging for children birth to age three who are more likely to be in private ECCE, and in private ECCE overall, given that these facilities may not have strong motivations to cooperate with government monitoring. This variation in types of ECCE inevitably leads to discrepancies in defining and measuring access to and participation in ECCE, between and even within countries (King et al., 2020). Taken collectively, this can contribute to substantial inaccuracies in estimates of access to quality ECCE, risking poor guidance for policymaking. The lack of clear definitions of ECCE-related services and outcomes creates a cycle in which key elements of ECCE are not defined, collected, or reported in similar ways, slowing the development, evaluation, and improvement of ECCE policy at the national level and prohibiting the comparison of ECCE systems across countries.

### Disentangling enrollment, attendance, and dosage

As explained earlier, the indicator for tracking children’s access to ECCE, “the participation rate in organized learning one year before primary school,” is calculated using two dimensions of participation: “net enrollment,^1^” or the ratio of the enrollment in early childhood education one year before the start of primary school, to the total population of children who are of the right age to attend (UNESCO, 2016), and “attendance,” (sometimes termed participation) or the percentage of children by age who report attending ECCE during the last year, reported through household surveys.

Enrollment and attendance data are often not clearly distinguished from each other in monitoring efforts. Available evidence on net enrollment rates suggests substantial miscounting of age-appropriate enrollment in ECCE and early primary grades. In some countries, children are either enrolling early in primary school or are enrolled in preprimary when they should be in primary grades, leading to inaccurate estimates of access to ECCE in the year before primary schooling (King et al., 2020). Further, as noted above, many types of ECCE—especially in the private sector—are not included in governmental counts, leading to substantial discrepancies between administrative and household data reported by parents (King, et al., 2020; Rao et al., 2021; Sincovich et al., 2019). Here, India is a case in point: “Despite the existence of multiple service providers, there are no reliable data available about the actual number of children attending ECCE provisions and their break down as per delivery services /type of services” (Ministry of Women & Child Development of India, 2013, p. 628). To highlight our point, we present data on reported gross enrollment ratios (GER) in several countries (see Table 3). The discrepancies between household surveys and administrative data are notable: one cannot assume that GER administrative data index the same information as caregiver-reported access to ECCE. These documented discrepancies support the need for closer collaboration and shared definitions between data collection efforts.

**Table 3:** Access to early childhood education and development programs reported through household surveys (HH) and gross enrollment percentages in preprimary reported through administrative data (Admin) by year for selected countries

	2015		2016		2017		2018		2019	
	HH	Admin	HH	Admin	HH	Admin	HH	Admin	HH	Admin
Bangladesh	.	32.36		35.52		41.74	.	40.82	77.45	.
Costa Rica	79.49	79.31	.	78.05	.	80.55	94.77	97.94	.	95.64
Ghana	.	120.86	82.36	119.02	86.50	116.79	88.23	114.55	.	117.01
Madagascar	.	18.02	.	28.51	82.97	37.56	59.36	39.61	.	40.15
Mongolia	.	79.23	.	82.65	.	85.35	84.26	86.67	.	.

Sources. MICS: Bangladesh Bureau of Statistics (BBS) and UNICEF Bangladesh. 2019; RSOC: India Ministry of Women and Child Development (MWCD) and UNICEF. 2014; DHS:; Gross enrollment rate (GER) and annual net enrollment rate (ANER) data were extracted from UNESCO UIS Stat http://data.uis.unesco.org/ on 17 June 2020 and November 2 2020

Interpretation of GER. “The GER for pre-primary education indicates a country’s theoretical capacity to accommodate children below the age when they start primary school education. A high value generally indicates a high degree of participation, regardless of children’s ages. Thus, it does not indicate the proportion of pre-primary age children actually enrolled (UNESCO Institute for Statistics, 2017), as some children may be over or under the official pre-primary age group. For example, if the official age group for pre-primary education in a country is ages 3 to 5, the GER will include children who are below 3 (underage) or above 6 years (overage).” Extracted from *Metadata for the global and thematic indicators for the follow-up and review of SDG 4 and Education 2030*. http://uis.unesco.org/sites/default/files/documents/metadata-global-thematic-indicators-sdg4-education2030-2017-en_1.pdf [uis.unesco.org] (accessed on 15 November 2017)

Critically, current national measurement of access to ECCE also does not take dosage into account, i.e., how much exposure children *actually* have to ECCE when enrolled (due to variation in program opening hours, children’s attendance and absenteeism rates, etc.). The global indicator limits reporting to ECCE access in the last year before the start of primary schooling but does not inquire as to the degree of participation during that year (nor does it acknowledge that additional years of ECCE could contribute positively to children’s learning). A recent analysis of evaluations of ECCE services provided to children aged birth to five years in the US found that only 38.2% reported information on dosage (Schindler et al., 2019), yet studies on dimensions of dosage, such as number of hours per day, show some associations with child learning and development (McCoy, et al., 2017; Rao et al., 2019) and also have implications for accommodation, staffing, and financing of ECCE policy and programs. The absence of information on dosage may considerably skew research findings on the role of ECCE in child development at a population level. Positive effects of a quality improvement initiative in public preschools in Chile on language and literacy outcomes occurred only for those children with the highest attendance (Arbour et al., 2016). Here, attendance was determined through unannounced observations during 15 randomly selected days of the preschool year. The 20 to 25% absenteeism rates in public preschools found in this study (whether considered on a typical day, or within child across one year) spurred a national campaign to address this participation problem (Fundación Educacional Oportunidad, 2020).

A similar phenomenon has been reported in the empirical literature focusing on primary school absenteeism: failing to understand whether absenteeism is excused or unexcused confounds conclusions regarding the relationship between absenteeism and academic outcomes (Gottfried, 2009). Indeed, household survey items that are used to generate global data on ECCE attendance are quite limited. For example, as noted in Table 2, the UNICEF-supported MICS generates data on access to ECCE by asking respondents about children’s attendance in early childhood programs during the current and previous years (UNICEF, 2021), which can mask highly variable participation rates (and program types). A “yes” response can indicate multiple scenarios: a “yes” due to perceived social desirability for their child to attend when in fact that child does not attend, the child is enrolled but rarely attends, the child attends a three-hour program one day per week, or the child attends from 6.30 am to 6.30 pm, five days per week. While each of these examples would be coded as “attends,” the dosage varies dramatically and impacts upon analytical precision with consequences for targeted policy decisions.

Obtaining more in-depth information on children’s participation in ECCE, including greater detail regarding what type of program the child has attended and the average number of hours per day/week of attendance, is thus critical to gaining a better understanding of children’s learning experiences. Although there are challenges for including clear and understandable questions in multi-topic household surveys, there is evidence to suggest that dosage can be measured reliably using household surveys (e.g., McCoy et al., 2017). Additional information on the details of the programs could provide greater insight into how frequently children attend and the extent of absenteeism, and building on points made in the previous section, whether the parent perceives the program as having an educational focus.

## Global monitoring of ECCE: Current status of data on quality in tandem with access

Equitable access may productively be defined as *access to and participation in quality ECCE for all children* (Britto et al., 2011). Reflecting national commitment to addressing both quality and access, several countries have invested in studies of quality and child development intended to inform policy and to serve as the basis for long-term efforts to improve ECCE quality. For example, in 2019, the Ministry of Education of Peru measured process quality and early child development nationally in two separate public systems of ECCE as well as a sample of private programs (Ministerio de Educación, 2019) and has committed to another assessment in 2023. Similar national-level studies of ECCE quality in public and private facilities have been conducted in several African countries, including Tanzania (UNICEF, 2019), Ethiopia (Rossiter et al., 2018), Egypt (Krafft et al., under review), and Liberia (Oxford Policy Management, 2018), as well as in Asian countries, including Bhutan (UNICEF, 2020a).

Several of these studies have been conducted as part of national monitoring initiatives, using nationally representative samples of ECCE centers (see Maldonado-Carreño et al., 2022, on Colombia; Raikes et al., 2020 on a sub-Saharan African country; each of these national studies showed some evidence of validity related to direct measures of child development). Among these, a few countries have committed to monitoring quality of ECCE together with direct measurement of children’s development in multiple domains more than once (e.g., every two years in Peru; Ministerio de Educación de Peru, 2019; South Africa’s recent initiative focused on a national census of early childhood; Egypt’s recent effort to institute national child development monitoring). A recent meta-analytic review shows that studies of observed process quality in ECCE in low- and middle-income countries show overall evidence of associations of higher quality with early development outcomes, with small to moderate magnitude of associations depending on the domain of outcome (Von Suchodoletz et al., 2022). Findings from these studies lend support to the idea that measuring ECCE quality at scale can and should be a key component of national measurement of Target 4.2.

At the same time, there are several complexities in defining and measuring quality at scale. Below we outline barriers to defining and measuring access to and participation in quality ECCE accurately.

### Defining quality ECCE

Globally, hundreds of millions of children pass through school systems without mastering basic literacy and numeracy skills, especially in LMICs (World Bank, 2019). The Human Capital Index 2020 shows that across countries, before the pandemic struck, a child could expect to attain an average of 56% of their potential productivity as a future worker. However, a child born in a low-income country could expect to attain only 37% of their productivity. In contrast, for a child born in a high-income country, this figure is 70% (World Bank, 2021). Quality of education is the link between attending school and emerging from school with the skills and competencies required for development of human capital. Therefore, Target 4.2 must ensure measurement of both access *and* quality. Tracking access to ECCE without considering quality in ECCE can create an inaccurate view of the extent to which young children are receiving the support necessary to succeed in school.

Unfortunately, inherent challenges in measuring quality at scale mean that few data are presently available on global ECCE quality (UNICEF, 2020b). Two core components of quality have been posited as a way of defining quality: structural quality and process quality (Howes et al., 2008; Peisner-Feinberg et al., 2001). Together, structural quality and process quality incorporate the thousands of back-and-forth interactions between facilitators/teachers and young children every day, supported by adequate facilities and learning materials, that are associated with children’s development across cognitive, social-emotional, and motor domains (Britto et al., 2011; Rao & Sun, 2015). Structural quality includes qualifications of ECCE teachers, teacher–child ratios, curriculum, and physical environments. For both private and public provision, some aspects of structural quality are likely to be outside the immediate control of an early learning setting because they are influenced by government regulations, the presence or absence of an approved curriculum, financial resourcing (influencing access to physical space, equipment, and learning materials), and quality monitoring.

Process quality, on the other hand, refers to the quality of interactions within the classroom (between teachers, children, and their peers). Teacher–child interactions are considered the most important determinant of quality as they reflect the quality of the child’s experience and thus, to some extent, are influenced by structural quality: a higher teacher–child ratio increases the likelihood of more frequent adult–child interactions (UNESCO, 2006). Research suggests that structural quality influences process quality and in turn child outcomes (Hong et al., 2019). To contribute positively to children’s development, both structural and process quality must be addressed. ECCE must meet basic quality standards, such as having trained, engaging and emotionally supportive teachers and facilitators, clear learning objectives, and access to materials (MELQO, 2017; Rao & Sun, 2015). Based on this research, teacher/child ratios, minimum teacher educational levels, and availability and engagement in professional development activities have all been identified as possible indicators of quality across countries (e.g., Montie et al., 2006).

The concepts of process and structural quality underlie several measurement tools (including the Environmental Ratings Scales (e.g., Harms et al., 1998); the TIPPS (Seidman et al., 2013); MELE (UNESCO, 2017a, 2017b); TEACH ECE (World Bank, 2021); and CLASS (Pianta, et al., 2008)). However, despite frequent reliance on existing quality definitions and measures, there are several limitations. While optimal practice would include the simultaneous monitoring of childhood development and the quality of provision across all types of ECCE (UNESCO, 2017a, 2017b), existing process quality measures largely rely on situation-based observational methods. These are typically resource intensive because training observers in the use of validated, observational instruments is costly and time consuming. A further challenge arising from observation-based measures is that while an instrument itself may be reliable, inter-rater reliability is also crucial to provide confidence in the data. For many measures, this requires extensive training to achieve and may not be feasible when taken to scale (e.g., Burchinal, 2018). Second, current widely used measures of quality are intended for centers serving children in the preschool years than for the wide range of home-, group-, and center-based care settings for infants and toddlers (for an exception, see Lopez Boo et al., 2016 in Ecuador). As a first step, using observational tools requires that all facilities are known by the government and agree to visits with outside observers, which is not a given especially for private providers (who are more frequently serving children birth to age three) who may have little incentive to take part in government monitoring (formal incentives are not usually provided for providers to participation national monitoring studies). Finally, there is little if any current evidence to suggest that observational quality measures meet psychometric standards of invariance or incorporate context variability for comparability across countries. Consequently, structural quality indicators, such as teacher qualifications and teacher–child ratios, are more visible and thus generally used as proxies for quality in large studies and in global comparisons of ECCE across countries (e.g., OECD’s Starting Strong). Shifting the focus to process quality indicators is a necessary step in ensuring that ECCE investments pay off for young children’s learning because in the absence of good measures of process quality, policy focus is likely to be too concentrated on structural quality. The EU has advanced in this regard, with the majority of EU countries’ national regulations for ECE incorporating attention to both structural and process quality (for programs serving children 3 years and older; European Commission, 2019).

Importantly, cultural and contextual conditions have a profound impact on how “quality” is defined across countries, precluding the clear cross-cultural definition necessary to underpin globally comparable measurement (Dahlberg et al., 1999; Myers, 2006; Raikes et al., 2020; Rao & Sun, 2015). While there are aspects of quality that have been shown to have relevance across countries, as noted below, there are similarly deep concerns about the relevance and applicability of “quality” definitions across contexts, including the potential for globally imposed quality standards to undermine delivery of culturally responsive ECCE (e.g., Burchinal, 2018; Myers, 2006). Values and beliefs about children’s capacity for participation, for example, inform curricular definitions of quality across cultures, as an analysis by Phillips, Ritchie, and Adair showed across Australia, New Zealand, and the United States (Phillips et al., 2020). Few large-scale studies of quality in LMICs have been conducted from measures that were wholly developed within country (for exceptions see Kaul & Bhattacharjea, 2019 in India). Many have been adapted from measures initially used in HIC contexts. The reliance on definitions of quality and measures originating in a small number of high-income countries has created an evidence base that does not address the diversity in practices in ECCE and leads to limited insight on the scope and definition of “quality” across contexts. From a policy perspective, few countries allow for variation in definitions of process quality or curriculum in ECE based on cultural diversity within the nation. One exception is Colombia, which introduced a modality of ECD services in their national policy in which indigenous and remote populations have the autonomy to set their definitions of quality as well as budget culturally specific quality supports within the national quality standards framework (Motta & Yoshikawa, 2018). Another important example is New Zealand, which instituted national curricula for ECE based on the largest indigenous group in the country, the Maori, in the 1980s.

While consensus on the specific aspects of quality that are most critical for child development in various settings has not been reached, there is nonetheless a growing body of work documenting small but reliable associations between various aspects of quality of children’s learning environments and subsequent child development and learning in LMICs (McCoy & Wolf, 2018; Raikes et al., 2020; Rao & Sun, 2015; Su et al., 2021; 2021; Von Suchodoletz et al., 2022; Wolf et al., 2018, 2019). These studies highlight the importance of different components of quality, such as access to learning materials and effective pedagogy, and their associations with child development.

Further, while many observational quality measures were designed for formal, center-based early childhood settings, it is important to note that with adequate investments in quality, all types of ECCE could meet standards for promoting children’s development and ensuring readiness for primary school education. These include formal preprimary settings, informal community-based preschools, and childcare settings. Private childcare settings, whether fee-based or non-fee-based, may be an especially important part of the ECCE system. Often overlooked as an avenue for supporting children’s development, childcare settings may be the type of ECCE provision that parents prefer and find most accessible, especially when access to formal preprimary is limited. Relying on broad definitions of quality that are applicable to *all* forms of ECCE is an important building block in creating inclusive and effective early childhood systems.

A third approach to defining quality is to focus on goals for children’s learning and development as complement to, or in place of, specific quality goals. The design and implementation of Early Learning Development Standards (ELDS) is one pathway toward defining goals for early childhood settings, by articulating the child development outcomes that are desired for all children across all types of ECCE rather than attempting to define quality. The ELDS do not include emphasis on specific aspects of quality, but rather focus on children’s developmental stages and desired competencies. The introduction of these standards has facilitated clearer focus on professional development, increased legitimization of the early childhood workforce, and a stronger emphasis on improving the quality, access, and equity of early childhood services (Kagan et al., 2013). However, few efforts have specifically examined the role of ELDS in influencing ECCE quality, and ELDS do not typically provide direct guidance on defining and measuring quality in ECCE settings (UNICEF, 2017).

In summary, definitions of ECCE quality should be culturally and contextually relevant. Yet, there is emerging agreement on factors that index quality regardless of context, such as having developmentally appropriate materials, safety and security, interactional quality, and pedagogical approaches that are designed for young children. Please see Table 4 for a summary of themes raised in this section.

**Table 4:** Definitions, measurement options and issues in measuring ECCE quality

	Process quality	Structural quality	Quality as defined by child development outcomes
How “quality” is defined in this conceptualization	Quality of interactions, access to learning materials and activities within early childhood settings	Qualifications of teachers, ratios, curriculum, and physical environments	Children’s achievement of developmental goals or milestones
How to measure	Observational measures conducted by trained observers for 2–4 h, many of which were originally developed for use in research	Most structural indicators do not require observation. Government or program reporting on teachers, ratios, use of curricula and safety of environments	Teacher/parent report on children’s development or assessment of children by trained assessors using validated measurement tools
Ability to make cross-country comparisons	Although several measures show validity within multiple countries, ability to make formal cross-country comparisons is not yet established	Can be defined and measured in similar ways across countries, based on global definitions	Some measures intended for global monitoring in use or under development. Degree of cross-cultural applicability varies by individual measure
Applicability across types of early childhood care and education programs	For most used observational measures, applicability across public/private, home-based and center-based facilities is not yet established	With some adjustment for different types of settings (i.e., home-based vs. center-based), may be possible to report across types of ECCE	Applicable across settings, but cross-age comparisons (i.e., between younger and older children) attending different types of programs (such as preschool vs. infant/toddler programs) may not be possible
Advantages	Provides measures of quality most directly related to child outcomes	Requires least amount of resource to measure consistently if all programs can be identified and included	Maintains strong focus on achievement of goals for children’s development
Disadvantages	Requires extensive training and resources to measure process quality reliably; depends on willingness of programs to participate in government monitoring	May not provide enough information on ECCE quality to adequately inform policies and practices that most strongly predict child development	Provides little to no insight on what aspects of the environment need to change to promote children’s development

## Conclusion: Steps toward generating accurate and actionable global data on access to quality ECCE

Based on this overview, we now draw implications for next steps to increase the accuracy, cultural relevance, and policy utility of ECCE measurement and monitoring. To obtain an accurate picture of the extent to which ECCE systems may be effective in preparing children for school, it is necessary to have information on access to and participation in all types of quality ECCE, ideally defined and measured in similar ways across countries. Globally comparable data allow insight into ECCE system functioning across countries.

Overall, at a global level, we need to better define “access to quality ECCE” using empirical data to clarify characteristics of various types of effective ECCE programs, to define how long children should attend a program to count as having *participated*, and to define global expectations for ECCE quality standards, including the possibility that such expectations are based on the process by which standards are developed and implemented, rather than the content of the standards. Below we outline steps toward better ECCE data and measurement with action items across communities of researchers, national stakeholders, and global organizations.

First, researchers can help address gaps in knowledge by reviewing existing studies to clarify and document the types and characteristics of programs that promote young children’s development, especially in relation to dosage and participation. Researchers also can contribute to insight into effective monitoring systems, by generating new evidence and measurement tools, documenting approaches to implementing data and monitoring systems, and defining how these systems can help promote access to quality ECCE. Monitoring systems, though rarely studied, are potentially a critical element of ensuring high-quality ECCE and require more documentation and research, especially in LMICs (e.g., Yoshikawa et al., 2018). A study of six high-performing ECCE systems in high-income contexts indicated that each had ECCE systems that implemented program standards and monitoring mechanisms, and further, that data collected were used to improve the quality of ECCE and related policies (Kagan et al., 2019). To ensure equity in access to quality ECCE, data must include both private and public facilities. This requires maintaining accurate registries of facilities and allocating resources for the routine monitoring of all facilities, and both structural and process quality should be included. To measure process quality, there is a need for development of monitoring tools using psychometrically robust measures of process quality that are cost effective for use at scale, require minimal observer training, are straightforward to administer, and have low, if any, associated fees for use.

Second, governments can create and implement scientifically informed quality standards that are culturally relevant, such as prioritizing aspects of learning environments that are culturally valued, and include all types of ECCE, private/public, and for all ages. As noted above, many countries have already begun the process of developing quality standards. Wide stakeholder input into national quality measure development and administration may help ensure cultural relevance (Ponguta et al., 2019). Specific policy mechanisms can allow for variation in quality as conceptualized by different cultural communities, including language-minority, rural, indigenous, and other groups (Motta & Yoshikawa, 2018).

Structural and process quality data can drive systems improvement in two ways. First, in national samples, indicators at the item level can inform professional development systems by focusing attention on aspects of quality that may be weak (whether nationally or in particular regions of a country). Here, professional development may draw on learning materials that have been provided to ECCE programs, could address the benefits of providing children with a choice of activities, or emphasize basic safety and hygiene in infrastructure and practices, to name a few common items across quality tools that have been used at national levels. Second, quality tools could be applied to every program and the feedback used in the existing monitoring systems to encourage program improvement. This universal approach is less common but is in planning stages or has been used in predominantly high-income countries, such as the United Kingdom, Chile, Finland, Australia, and the United States.

Third, investing in infrastructure for ongoing national monitoring of ECCE program quality is essential, by national governments as well as international organizations. Depending on the status of country monitoring systems, this may require expanding the scope of monitoring systems to include all types of ECCE, regular data collection on indicators of quality and the number and ages of children who attend, and investments in technology to facilitate data collection, aggregation, and analyses.

Fourth, to leverage national and global momentum on measuring access to quality ECCE, global actors, such as UNESCO and UNICEF, can define and collect proxy indicators of access to quality ECCE. Recognizing the potential negative consequences of defining quality too narrowly, we propose possible criteria to monitor globally as indicators of quality ECCE: (1) the presence of national standards in the form of early learning standards/guidelines and/or a national curriculum/framework must be in place; (2) minimum qualifications for teachers must be outlined in government documents; (3) programs available at least 2 h per day for at least 100 days in a year to ensure adequate dosage; (4) a wide range of programs (including both private and public) must be recognized, registered, resourced, and monitored for improvement by the government; and (5) define indicators of structural quality to be collected at the country level, including teacher qualifications, class size, teacher–child ratio, and the physical environment, similar to approaches in primary and secondary education. The recent work by UNESCO and OECD to develop and test surveys on early childhood professionals in low- and middle-income countries, the Survey of Teachers in Preprimary Education (STEPP; UNESCO, 2020), provides evidence that surveys of teachers are feasible and can yield important insights on working conditions, teachers’ qualifications, and characteristics of classrooms.

While evidence is not yet available on the content of country-level ECCE quality standards, it is feasible to count the number of countries with quality standards in place, whether those standards are applicable to all types of ECCE, and whether all types of ECCE are routinely monitored by governments, as we propose above. Collected and aggregated across countries, these data could be instrumental in providing a more comprehensive and global view of access to quality ECCE through Target 4.2. Regional actors also have an important role in play in harnessing innovations from regions and creating opportunities for countries to learn from regional peers on best practices (for example, the African Association for the Development of Education; Early Childhood Development Action Network; the International Step by Step Association; Asia–Pacific Regional Network for Early Childhood; Arab Network for Early Childhood; and knowledge hubs hosted by the Inter-American Development Bank).

Fifth, questions on national household demographic surveys could be modified to collect better information on children’s ECCE dosage, again with leadership by both national governments and international organizations. Although research from the US has suggested that parent ratings of quality do not correspond with objective indicators (Bassok et al., 2018), additional work could experiment with questions on access to quality ECCE across countries. Improving the scope and precision of household surveys also plays a role in improving data on access to quality ECCE. At present, there is little guidance on how household surveys can best capture children’s access to quality ECCE. Better information on dosage would help, but more work is needed to assess how to make questions on parents’ perceptions of quality ECCE accurate and useful, especially questions relating to whether ECCE programs are perceived to have educational foci and whether parents feel confident that programs will help prepare children for school.

At present, few LMICs have the infrastructure available to engage in monitoring to produce information on access to quality ECCE (UNICEF, 2020a, 2020b), although many have quality standards in place and some form of ECCE monitoring underway to track access to quality ECCE across regions and income levels. Several efforts are underway in low- and middle-income countries to produce better data on early childhood systems. Preprimary education was incorporated into the Mexican school census starting in the early 2000s (Yoshikawa et al., 2007). Building on child development and quality standards already in place, South Africa has recently undertaken a national census of all early childhood facilities, public and private, that will include measurement of quality and child development, as well as information on children’s demographic and family characteristics and the early childhood workforce (Government of South Africa, 2021). This census will form the basis for long-term monitoring of all ECCE facilities.

Building effective state and national monitoring systems would not only improve the quality and accuracy of data used to inform policy but would also help move countries toward data-driven ECCE systems, encouraging accountability and allowing rigorous insight into ECCE system effectiveness. Ensuring that feedback loops systematically use data to drive improvement is critical (for example, links between program, local, and subnational level data on quality and access and each of these levels of implementation of ECCE workforce and governance systems; Pritchett, 2013).

We conclude with a cautionary note. Early childhood care and education by itself, even if implemented nationally with high quality, is not a panacea. Social mobility and reductions in societal inequality will require far more, including efforts to directly reduce poverty; address discrimination and marginalization of migrant, indigenous, language-minority, and other communities; and sustain investments in children’s learning and development through adolescence (Nalani et al., 2021). Early childhood systems in many countries are under-resourced and stressed, with limited resources available to invest in data and monitoring. Momentum to implement the Early Learning and Development Standards, for example, lagged when attempting to fully integrate standards into country processes (UNICEF, 2017). Yet within- and cross-country insights may yield valuable new ideas. Supporting countries to develop better mechanisms for assessing the functioning of ECCE systems may be a feasible starting point. While access to quality ECCE is only one part of the holistic, intersectoral approach that is needed to fully support young children’s development, attention to data on access to *quality* ECCE could lay the groundwork for more comprehensive monitoring systems that integrate health, social protection, and nutrition systems. Improving data on ECCE access will improve the ability to identify policy-relevant comparisons and build the infrastructure necessary for ongoing program and policy improvement.

## Data Availability

Not applicable.

## Footnotes

1 Gross enrollment refers to the total number of children enrolled and can be much higher than net enrollment due to under-age and over-age enrollment (King et al., 2020).
